# 3-(4-Methoxy­benz­yl)-2-methyl-1-phenyl­sulfonyl-1*H*-indole

**DOI:** 10.1107/S1600536809010046

**Published:** 2009-03-25

**Authors:** T. Kavitha, M. Thenmozhi, V. Dhayalan, A. K. Mohanakrishnan, M. N. Ponnuswamy

**Affiliations:** a Centre of Advanced Study in Crystallography and Biophysics, University of Madras, Guindy Campus, Chennai 600 025, India; b Department of Organic Chemistry, University of Madras, Guindy Campus, Chennai 600 025, India

## Abstract

There are two crystallographically independent mol­ecules in the asymmetric unit of the title compound, C_23_H_21_NO_3_S. The indole ring system is approximately perpendicular to the sulfonyl phenyl ring in both mol­ecules [dihedral angles = 85.42 (8) and 88.30 (9)°]. C—H⋯O inter­actions between mol­ecules stabilize the crystal structure.

## Related literature

For the Thorpe–Ingold effect, see: Bassindale (1984 ▶). For bond-length data, see: Allen *et al.* (1987 ▶). For the biological activity of sulfur-containing compounds, see: De-Benedetti *et al*, (1985 ▶); Krishnaiah *et al.* (1995 ▶).
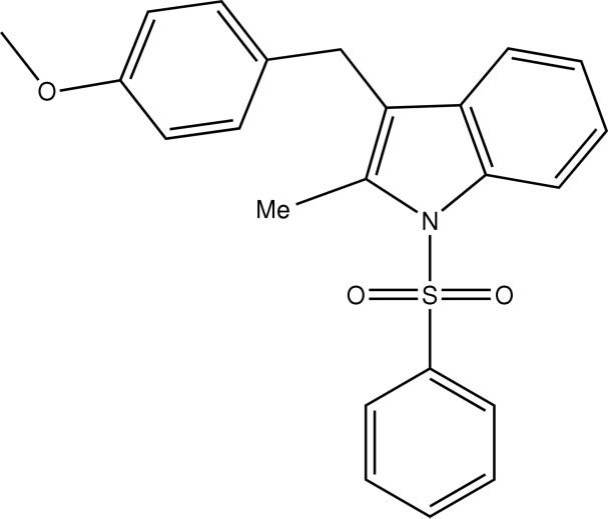


## Experimental

### 

#### Crystal data

C_23_H_21_NO_3_S
*M* *_r_* = 391.47
Triclinic, 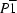

*a* = 11.4366 (4) Å
*b* = 13.6021 (5) Å
*c* = 14.0001 (5) Å
α = 74.154 (1)°
β = 67.773 (2)°
γ = 88.848 (2)°
*V* = 1930.96 (12) Å^3^
*Z* = 4
Mo *K*α radiation
μ = 0.19 mm^−1^
*T* = 293 K
0.20 × 0.20 × 0.18 mm

#### Data collection

Bruker Kappa APEXII diffractometer
Absorption correction: multi-scan (*SADABS*; Sheldrick, 2001 ▶) *T*_min_ = 0.963, *T*_max_ = 0.970
45568 measured reflections
10459 independent reflections
7561 reflections with *I* > 2σ(*I*)
*R*_int_ = 0.029

#### Refinement

*R*[*F* ^2^ > 2σ(*F* ^2^)] = 0.043
*wR*(*F* ^2^) = 0.140
*S* = 1.07
10459 reflections
509 parameters
H-atom parameters constrained
Δρ_max_ = 0.26 e Å^−3^
Δρ_min_ = −0.29 e Å^−3^

### 

Data collection: *APEX2* (Bruker, 2004 ▶); cell refinement: *SAINT* (Bruker, 2004 ▶); data reduction: *SAINT*; program(s) used to solve structure: *SHELXS97* (Sheldrick, 2008 ▶); program(s) used to refine structure: *SHELXL97* (Sheldrick, 2008 ▶); molecular graphics: *ORTEP-3* (Farrugia, 1997 ▶); software used to prepare material for publication: *SHELXL97* and *PLATON* (Spek, 2009 ▶).

## Supplementary Material

Crystal structure: contains datablocks I, global. DOI: 10.1107/S1600536809010046/bt2902sup1.cif

Structure factors: contains datablocks I. DOI: 10.1107/S1600536809010046/bt2902Isup2.hkl

Additional supplementary materials:  crystallographic information; 3D view; checkCIF report

## Figures and Tables

**Table 1:** Hydrogen-bond geometry (Å, °)

*D*—H⋯*A*	*D*—H	H⋯*A*	*D*⋯*A*	*D*—H⋯*A*
C11*A*—H11*A*⋯O1*B*^i^	0.93	2.52	3.397 (2)	157
C11*B*—H11*B*⋯O1*A*^ii^	0.93	2.49	3.337 (2)	151
C15*A*—H15*A*⋯O3*B*^iii^	0.93	2.53	3.398 (2)	155

Symmetry codes: (i) 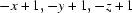; (ii) 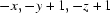; (iii) 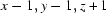.

**Fig. 1.**
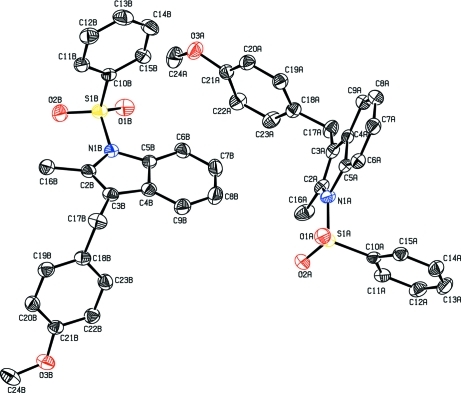
ORTEP plot of the molecule showing that the thermal ellipsoids are drawn at 20% probability level. H atoms have been omitted for clarity.

**Fig. 2.**
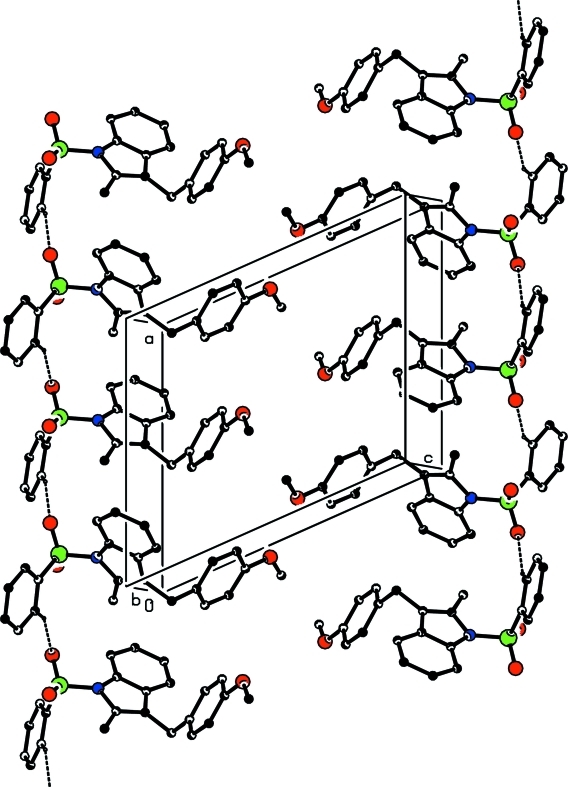
The molecular packing of the compound viewed down the a axis is shown. Dashed lines indicate hydrogen bonds.

## supplementary crystallographic information

### Comment

Many sulfur-containing compounds, such as sulfates, sulfones and sulfonamides, exhibit insecticidal, germicidal, antimicrobial and antibacterial activities (De-Benedetti *et al.*, 1985; Krishnaiah *et al.*, 1995).

The asymmetric unit contains two crystallographically independent molecules, A and B (Fig. 1). As a result of the electron-withdrawing character of the phenylsulfonyl group, the bond lengths N1—C2 [1.4268 (24) Å and 1.4315 (25) Å] and N1—C5 [1.4180 (23) Å and 1.4160 (24) Å] in molecules A and B are longer than the mean value of 1.355 (14) Å (Allen *et al.*, 1987). Atom S has a tetrahedral geometry, with the O—S—O and N—S—C angles deviating significantly from ideal value, and this may be attributed to the Thorpe-Ingold effect (Bassindale, 1984). The mean plane of the planar indole ring system and the sulfonyl bound phenyl ring are approximately perpendicular to each other in both the molecules A [85.41 (8)°] and B [88.30 (9)°]. The benzylphenyl rings are oriented at angles of 66.73 (8)° and 71.93 (8)° in molecules A & B, respectively.

In the crystal structure, C—H···O interactions (Table 2) link the molecules into linear chain extending along the *a* axis.

### Experimental

1-Phenylsulfonyl-2-methyl-3-bromomethylindole (2 g, 5.49 mmol) was dissolved in dry CH_3_CN (20 ml). To this anisole (0.89 ml, 8.23 mmol), ZnBr_2_ (2.47 g, 10.96 mmol) were added and refluxed for 12 h. The reaction mixture was quenched with ice containing few drops of conc. HCl, extracted with CHCl_3_ (3 × 5 ml) and dried Na_2_SO_4_. The solvent was removed under vacuo. Then crude was recrystallized from CDCl_3_ to get the diffraction quality crystals.

### Refinement

H atoms were positioned geometrically (C—H = 0.93–0.98 Å) and allowed to ride on their parent atoms, with *U*_iso_(H) = 1.5*U*_eq_(C_methyl_) and 1.2*U*_eq_(C).

### Crystal data

**Table:**

C_23_H_21_NO_3_S	*Z* = 4
*M**_r_* = 391.47	*F*(000) = 824
Triclinic, *P*1	*D*_x_ = 1.347 Mg m^−^^3^
Hall symbol: -P 1	Mo *K*α radiation, λ = 0.71073 Å
*a* = 11.4366 (4) Å	Cell parameters from 10459 reflections
*b* = 13.6021 (5) Å	θ = 1.6–29.3°
*c* = 14.0001 (5) Å	µ = 0.19 mm^−^^1^
α = 74.154 (1)°	*T* = 293 K
β = 67.773 (2)°	Block, white
γ = 88.848 (2)°	0.20 × 0.20 × 0.18 mm
*V* = 1930.96 (12) Å^3^	

### Data collection

**Table:**

Bruker Kappa APEXII diffractometer	10459 independent reflections
Radiation source: fine-focus sealed tube	7561 reflections with *I* > 2σ(*I*)
graphite	*R*_int_ = 0.029
ω and φ scans	θ_max_ = 29.3°, θ_min_ = 1.6°
Absorption correction: multi-scan (*SADABS*; Sheldrick, 2001)	*h* = −15→15
*T*_min_ = 0.963, *T*_max_ = 0.970	*k* = −18→18
45568 measured reflections	*l* = −19→19

### Refinement

**Table:**

Refinement on *F*^2^	Primary atom site location: structure-invariant direct methods
Least-squares matrix: full	Secondary atom site location: difference Fourier map
*R*[*F*^2^ > 2σ(*F*^2^)] = 0.043	Hydrogen site location: inferred from neighbouring sites
*wR*(*F*^2^) = 0.140	H-atom parameters constrained
*S* = 1.07	*w* = 1/[σ^2^(*F*_o_^2^) + (0.0656*P*)^2^ + 0.438*P*] where *P* = (*F*_o_^2^ + 2*F*_c_^2^)/3
10459 reflections	(Δ/σ)_max_ = 0.017
509 parameters	Δρ_max_ = 0.26 e Å^−^^3^
0 restraints	Δρ_min_ = −0.29 e Å^−^^3^

### Special details

**Table:**

Geometry. All e.s.d.'s (except the e.s.d. in the dihedral angle between two l.s. planes) are estimated using the full covariance matrix. The cell e.s.d.'s are taken into account individually in the estimation of e.s.d.'s in distances, angles and torsion angles; correlations between e.s.d.'s in cell parameters are only used when they are defined by crystal symmetry. An approximate (isotropic) treatment of cell e.s.d.'s is used for estimating e.s.d.'s involving l.s. planes.
Refinement. Refinement of *F*^2^ against ALL reflections. The weighted *R*-factor *wR* and goodness of fit *S* are based on *F*^2^, conventional *R*-factors *R* are based on *F*, with *F* set to zero for negative *F*^2^. The threshold expression of *F*^2^ > σ(*F*^2^) is used only for calculating *R*-factors(gt) *etc*. and is not relevant to the choice of reflections for refinement. *R*-factors based on *F*^2^ are statistically about twice as large as those based on *F*, and *R*- factors based on ALL data will be even larger.

### Fractional atomic coordinates and isotropic or equivalent isotropic displacement parameters (Å^2^)

**Table:**

	*x*	*y*	*z*	*U*_iso_*/*U*_eq_	
N1A	−0.17038 (13)	0.14863 (11)	1.12497 (11)	0.0443 (3)	
C2A	−0.05288 (16)	0.20836 (13)	1.05543 (14)	0.0466 (4)	
C3A	−0.00977 (16)	0.18342 (13)	0.96228 (13)	0.0454 (4)	
C4A	−0.09665 (15)	0.10399 (12)	0.97011 (13)	0.0424 (3)	
C5A	−0.19475 (15)	0.08125 (12)	1.07183 (13)	0.0405 (3)	
C6A	−0.29250 (17)	0.00611 (13)	1.10475 (14)	0.0480 (4)	
H6A	−0.3569	−0.0088	1.1730	0.058*	
C7A	−0.2910 (2)	−0.04618 (14)	1.03236 (17)	0.0561 (4)	
H7A	−0.3559	−0.0970	1.0521	0.067*	
C8A	−0.1947 (2)	−0.02427 (15)	0.93107 (17)	0.0604 (5)	
H8A	−0.1958	−0.0607	0.8840	0.072*	
C9A	−0.09766 (19)	0.05022 (14)	0.89889 (15)	0.0531 (4)	
H9A	−0.0335	0.0646	0.8305	0.064*	
C10A	−0.17349 (15)	0.03061 (12)	1.31777 (11)	0.0402 (3)	
C11A	−0.06498 (17)	0.05180 (15)	1.33280 (14)	0.0506 (4)	
H11A	−0.0277	0.1187	1.3096	0.061*	
C12A	−0.0135 (2)	−0.02814 (18)	1.38270 (16)	0.0625 (5)	
H12A	0.0597	−0.0154	1.3932	0.075*	
C13A	−0.0695 (2)	−0.12693 (18)	1.41720 (16)	0.0658 (6)	
H13A	−0.0340	−0.1804	1.4511	0.079*	
C14A	−0.1772 (2)	−0.14712 (15)	1.40197 (15)	0.0593 (5)	
H14A	−0.2142	−0.2141	1.4254	0.071*	
C15A	−0.23062 (16)	−0.06838 (13)	1.35205 (13)	0.0463 (4)	
H15A	−0.3037	−0.0815	1.3416	0.056*	
C16A	0.0059 (2)	0.28689 (17)	1.08363 (18)	0.0683 (6)	
H16A	0.0819	0.3200	1.0235	0.102*	
H16B	0.0265	0.2544	1.1446	0.102*	
H16C	−0.0526	0.3370	1.1010	0.102*	
C17A	0.11050 (17)	0.22932 (15)	0.86645 (15)	0.0566 (5)	
H17A	0.1747	0.2425	0.8922	0.068*	
H17B	0.1401	0.1792	0.8270	0.068*	
C18A	0.09861 (16)	0.32724 (14)	0.79008 (14)	0.0478 (4)	
C19A	0.02228 (18)	0.32887 (14)	0.73256 (15)	0.0525 (4)	
H19A	−0.0261	0.2692	0.7448	0.063*	
C20A	0.01679 (19)	0.41685 (14)	0.65795 (16)	0.0549 (4)	
H20A	−0.0348	0.4162	0.6203	0.066*	
C21A	0.08779 (17)	0.50612 (13)	0.63898 (14)	0.0479 (4)	
C22A	0.15963 (18)	0.50785 (15)	0.69830 (16)	0.0575 (5)	
H22A	0.2043	0.5685	0.6887	0.069*	
C23A	0.16458 (18)	0.41830 (16)	0.77235 (16)	0.0578 (5)	
H23A	0.2142	0.4197	0.8115	0.069*	
C24A	0.1586 (2)	0.67909 (16)	0.5325 (2)	0.0744 (6)	
H24A	0.2452	0.6634	0.5143	0.112*	
H24B	0.1332	0.7072	0.5924	0.112*	
H24C	0.1508	0.7282	0.4718	0.112*	
O1A	−0.37088 (12)	0.09840 (11)	1.28435 (10)	0.0568 (3)	
O2A	−0.21330 (15)	0.22300 (10)	1.27677 (11)	0.0620 (4)	
O3A	0.07983 (15)	0.58831 (10)	0.56047 (12)	0.0646 (4)	
S1A	−0.24227 (4)	0.13171 (3)	1.25612 (3)	0.04469 (11)	
N1B	0.69123 (14)	0.70806 (11)	−0.13816 (11)	0.0464 (3)	
C2B	0.57402 (17)	0.73507 (14)	−0.06982 (14)	0.0476 (4)	
C3B	0.52747 (16)	0.65959 (14)	0.02341 (14)	0.0477 (4)	
C4B	0.61181 (17)	0.58004 (13)	0.01765 (14)	0.0472 (4)	
C5B	0.71146 (16)	0.60928 (13)	−0.08345 (14)	0.0444 (4)	
C6B	0.80826 (19)	0.54755 (15)	−0.11375 (17)	0.0550 (4)	
H6B	0.8739	0.5672	−0.1815	0.066*	
C7B	0.8031 (2)	0.45559 (16)	−0.0391 (2)	0.0670 (6)	
H7B	0.8668	0.4123	−0.0570	0.080*	
C8B	0.7064 (3)	0.42618 (16)	0.0611 (2)	0.0722 (6)	
H8B	0.7063	0.3637	0.1094	0.087*	
C9B	0.6102 (2)	0.48702 (15)	0.09113 (17)	0.0621 (5)	
H9B	0.5453	0.4666	0.1592	0.075*	
C10B	0.68658 (15)	0.69410 (13)	−0.32679 (12)	0.0419 (3)	
C11B	0.57731 (17)	0.72794 (15)	−0.33924 (14)	0.0510 (4)	
H11B	0.5433	0.7846	−0.3174	0.061*	
C12B	0.5192 (2)	0.67717 (18)	−0.38412 (18)	0.0658 (5)	
H12B	0.4453	0.6993	−0.3928	0.079*	
C13B	0.5700 (2)	0.59387 (19)	−0.41614 (19)	0.0727 (6)	
H13B	0.5303	0.5595	−0.4463	0.087*	
C14B	0.6788 (2)	0.56071 (18)	−0.40416 (18)	0.0699 (6)	
H14B	0.7123	0.5041	−0.4263	0.084*	
C15B	0.73916 (19)	0.61057 (15)	−0.35946 (15)	0.0548 (4)	
H15B	0.8133	0.5885	−0.3515	0.066*	
C16B	0.5195 (2)	0.83354 (18)	−0.09892 (18)	0.0675 (6)	
H16D	0.4443	0.8374	−0.0390	0.101*	
H16E	0.4985	0.8370	−0.1599	0.101*	
H16F	0.5806	0.8898	−0.1167	0.101*	
C17B	0.40745 (18)	0.65872 (17)	0.11788 (16)	0.0598 (5)	
H17C	0.3768	0.5881	0.1600	0.072*	
H17D	0.3436	0.6879	0.0914	0.072*	
C18B	0.42235 (16)	0.71719 (14)	0.19031 (14)	0.0488 (4)	
C19B	0.35364 (17)	0.79902 (16)	0.20903 (15)	0.0552 (4)	
H19B	0.2979	0.8195	0.1747	0.066*	
C20B	0.36479 (18)	0.85184 (15)	0.27728 (15)	0.0543 (4)	
H20B	0.3178	0.9073	0.2879	0.065*	
C21B	0.44567 (17)	0.82183 (13)	0.32917 (13)	0.0472 (4)	
C22B	0.51781 (18)	0.74128 (15)	0.30985 (15)	0.0530 (4)	
H22B	0.5749	0.7221	0.3429	0.064*	
C23B	0.50558 (17)	0.68950 (14)	0.24221 (16)	0.0534 (4)	
H23B	0.5538	0.6348	0.2308	0.064*	
C24B	0.3822 (2)	0.94124 (19)	0.43162 (19)	0.0734 (6)	
H24D	0.2957	0.9115	0.4661	0.110*	
H24E	0.3915	0.9970	0.3692	0.110*	
H24F	0.4043	0.9663	0.4809	0.110*	
O1B	0.88915 (12)	0.73377 (12)	−0.29917 (11)	0.0627 (4)	
O2B	0.73674 (15)	0.86335 (10)	−0.29267 (12)	0.0632 (4)	
O3B	0.46289 (14)	0.86603 (12)	0.40024 (12)	0.0656 (4)	
S1B	0.76171 (4)	0.75867 (3)	−0.26991 (3)	0.04760 (12)	

### Atomic displacement parameters (Å^2^)

**Table:**

	*U*^11^	*U*^22^	*U*^33^	*U*^12^	*U*^13^	*U*^23^
N1A	0.0514 (8)	0.0460 (7)	0.0356 (7)	0.0003 (6)	−0.0189 (6)	−0.0089 (6)
C2A	0.0511 (9)	0.0450 (9)	0.0435 (9)	0.0003 (7)	−0.0261 (7)	−0.0011 (7)
C3A	0.0432 (8)	0.0452 (9)	0.0431 (9)	0.0070 (7)	−0.0191 (7)	−0.0021 (7)
C4A	0.0449 (8)	0.0406 (8)	0.0411 (8)	0.0119 (7)	−0.0190 (7)	−0.0080 (6)
C5A	0.0462 (8)	0.0402 (8)	0.0385 (8)	0.0092 (6)	−0.0219 (7)	−0.0090 (6)
C6A	0.0493 (9)	0.0460 (9)	0.0466 (9)	0.0026 (7)	−0.0198 (7)	−0.0081 (7)
C7A	0.0659 (12)	0.0457 (9)	0.0635 (12)	0.0020 (8)	−0.0337 (10)	−0.0135 (8)
C8A	0.0823 (14)	0.0525 (10)	0.0584 (11)	0.0132 (10)	−0.0346 (11)	−0.0247 (9)
C9A	0.0631 (11)	0.0506 (10)	0.0447 (9)	0.0145 (8)	−0.0185 (8)	−0.0161 (8)
C10A	0.0430 (8)	0.0478 (9)	0.0277 (7)	0.0072 (7)	−0.0109 (6)	−0.0120 (6)
C11A	0.0520 (10)	0.0601 (11)	0.0414 (9)	0.0046 (8)	−0.0189 (7)	−0.0163 (8)
C12A	0.0565 (11)	0.0848 (15)	0.0520 (11)	0.0137 (10)	−0.0292 (9)	−0.0171 (10)
C13A	0.0696 (13)	0.0728 (14)	0.0492 (11)	0.0231 (11)	−0.0267 (10)	−0.0043 (9)
C14A	0.0681 (12)	0.0515 (10)	0.0433 (9)	0.0059 (9)	−0.0136 (9)	−0.0016 (8)
C15A	0.0454 (9)	0.0519 (9)	0.0361 (8)	0.0036 (7)	−0.0115 (7)	−0.0101 (7)
C16A	0.0794 (14)	0.0687 (13)	0.0589 (12)	−0.0191 (11)	−0.0371 (11)	−0.0049 (10)
C17A	0.0441 (9)	0.0582 (11)	0.0525 (10)	0.0067 (8)	−0.0140 (8)	0.0005 (8)
C18A	0.0403 (8)	0.0527 (10)	0.0405 (8)	0.0029 (7)	−0.0101 (7)	−0.0058 (7)
C19A	0.0583 (10)	0.0451 (9)	0.0539 (10)	−0.0014 (8)	−0.0237 (8)	−0.0106 (8)
C20A	0.0641 (11)	0.0538 (10)	0.0548 (10)	0.0045 (9)	−0.0321 (9)	−0.0147 (8)
C21A	0.0503 (9)	0.0454 (9)	0.0428 (9)	0.0052 (7)	−0.0141 (7)	−0.0106 (7)
C22A	0.0549 (10)	0.0527 (10)	0.0586 (11)	−0.0101 (8)	−0.0213 (9)	−0.0064 (8)
C23A	0.0505 (10)	0.0658 (12)	0.0554 (11)	−0.0063 (9)	−0.0265 (9)	−0.0052 (9)
C24A	0.0725 (14)	0.0504 (11)	0.0785 (15)	−0.0012 (10)	−0.0214 (12)	0.0043 (10)
O1A	0.0454 (7)	0.0710 (9)	0.0518 (7)	0.0162 (6)	−0.0166 (6)	−0.0182 (6)
O2A	0.0857 (10)	0.0498 (7)	0.0597 (8)	0.0162 (7)	−0.0311 (7)	−0.0263 (6)
O3A	0.0790 (9)	0.0495 (7)	0.0606 (8)	0.0022 (7)	−0.0321 (7)	−0.0012 (6)
S1A	0.0505 (2)	0.0463 (2)	0.0390 (2)	0.01163 (17)	−0.01780 (17)	−0.01493 (16)
N1B	0.0526 (8)	0.0511 (8)	0.0422 (7)	0.0124 (6)	−0.0233 (6)	−0.0175 (6)
C2B	0.0517 (9)	0.0569 (10)	0.0483 (9)	0.0147 (8)	−0.0282 (8)	−0.0251 (8)
C3B	0.0480 (9)	0.0562 (10)	0.0476 (9)	0.0040 (7)	−0.0222 (7)	−0.0230 (8)
C4B	0.0534 (9)	0.0468 (9)	0.0490 (9)	0.0009 (7)	−0.0256 (8)	−0.0173 (7)
C5B	0.0511 (9)	0.0459 (9)	0.0484 (9)	0.0071 (7)	−0.0293 (8)	−0.0185 (7)
C6B	0.0573 (10)	0.0612 (11)	0.0608 (11)	0.0167 (9)	−0.0310 (9)	−0.0287 (9)
C7B	0.0820 (14)	0.0573 (12)	0.0873 (16)	0.0295 (11)	−0.0520 (13)	−0.0342 (11)
C8B	0.1045 (18)	0.0479 (11)	0.0780 (15)	0.0154 (11)	−0.0527 (14)	−0.0150 (10)
C9B	0.0813 (14)	0.0501 (10)	0.0571 (11)	−0.0019 (10)	−0.0320 (10)	−0.0107 (9)
C10B	0.0437 (8)	0.0461 (8)	0.0327 (7)	0.0005 (7)	−0.0122 (6)	−0.0100 (6)
C11B	0.0528 (10)	0.0558 (10)	0.0466 (9)	0.0073 (8)	−0.0210 (8)	−0.0157 (8)
C12B	0.0623 (12)	0.0802 (14)	0.0649 (13)	0.0040 (10)	−0.0337 (10)	−0.0230 (11)
C13B	0.0859 (16)	0.0802 (15)	0.0667 (13)	−0.0046 (12)	−0.0369 (12)	−0.0324 (12)
C14B	0.0872 (16)	0.0659 (13)	0.0640 (13)	0.0103 (11)	−0.0261 (12)	−0.0352 (11)
C15B	0.0572 (10)	0.0589 (11)	0.0484 (10)	0.0116 (9)	−0.0176 (8)	−0.0201 (8)
C16B	0.0811 (14)	0.0768 (14)	0.0602 (12)	0.0369 (12)	−0.0388 (11)	−0.0296 (11)
C17B	0.0474 (10)	0.0760 (13)	0.0584 (11)	−0.0003 (9)	−0.0168 (9)	−0.0281 (10)
C18B	0.0410 (8)	0.0593 (10)	0.0421 (9)	0.0012 (7)	−0.0108 (7)	−0.0157 (8)
C19B	0.0485 (9)	0.0699 (12)	0.0487 (10)	0.0126 (9)	−0.0202 (8)	−0.0176 (9)
C20B	0.0525 (10)	0.0555 (10)	0.0496 (10)	0.0127 (8)	−0.0139 (8)	−0.0156 (8)
C21B	0.0476 (9)	0.0491 (9)	0.0382 (8)	−0.0023 (7)	−0.0105 (7)	−0.0104 (7)
C22B	0.0511 (10)	0.0589 (11)	0.0530 (10)	0.0079 (8)	−0.0243 (8)	−0.0163 (8)
C23B	0.0520 (10)	0.0530 (10)	0.0575 (11)	0.0117 (8)	−0.0212 (8)	−0.0201 (8)
C24B	0.0702 (13)	0.0791 (15)	0.0656 (13)	0.0018 (11)	−0.0072 (11)	−0.0397 (12)
O1B	0.0448 (7)	0.0811 (10)	0.0611 (8)	−0.0019 (6)	−0.0182 (6)	−0.0214 (7)
O2B	0.0816 (10)	0.0444 (7)	0.0637 (8)	−0.0023 (6)	−0.0316 (7)	−0.0104 (6)
O3B	0.0721 (9)	0.0721 (9)	0.0626 (9)	0.0086 (7)	−0.0270 (7)	−0.0338 (7)
S1B	0.0492 (2)	0.0500 (2)	0.0440 (2)	−0.00122 (18)	−0.01884 (18)	−0.01247 (18)

### Geometric parameters (Å, °)

**Table:**

N1A—C5A	1.418 (2)	N1B—C5B	1.416 (2)
N1A—C2A	1.427 (2)	N1B—C2B	1.432 (2)
N1A—S1A	1.6538 (14)	N1B—S1B	1.6522 (15)
C2A—C3A	1.344 (2)	C2B—C3B	1.344 (3)
C2A—C16A	1.487 (3)	C2B—C16B	1.486 (3)
C3A—C4A	1.436 (2)	C3B—C4B	1.433 (2)
C3A—C17A	1.504 (2)	C3B—C17B	1.501 (3)
C4A—C9A	1.392 (2)	C4B—C9B	1.391 (3)
C4A—C5A	1.395 (2)	C4B—C5B	1.397 (3)
C5A—C6A	1.381 (2)	C5B—C6B	1.383 (2)
C6A—C7A	1.383 (3)	C6B—C7B	1.379 (3)
C6A—H6A	0.9300	C6B—H6B	0.9300
C7A—C8A	1.383 (3)	C7B—C8B	1.374 (4)
C7A—H7A	0.9300	C7B—H7B	0.9300
C8A—C9A	1.371 (3)	C8B—C9B	1.370 (3)
C8A—H8A	0.9300	C8B—H8B	0.9300
C9A—H9A	0.9300	C9B—H9B	0.9300
C10A—C11A	1.383 (2)	C10B—C11B	1.379 (2)
C10A—C15A	1.384 (2)	C10B—C15B	1.382 (2)
C10A—S1A	1.7541 (16)	C10B—S1B	1.7531 (17)
C11A—C12A	1.374 (3)	C11B—C12B	1.373 (3)
C11A—H11A	0.9300	C11B—H11B	0.9300
C12A—C13A	1.377 (3)	C12B—C13B	1.370 (3)
C12A—H12A	0.9300	C12B—H12B	0.9300
C13A—C14A	1.372 (3)	C13B—C14B	1.368 (3)
C13A—H13A	0.9300	C13B—H13B	0.9300
C14A—C15A	1.377 (3)	C14B—C15B	1.381 (3)
C14A—H14A	0.9300	C14B—H14B	0.9300
C15A—H15A	0.9300	C15B—H15B	0.9300
C16A—H16A	0.9600	C16B—H16D	0.9600
C16A—H16B	0.9600	C16B—H16E	0.9600
C16A—H16C	0.9600	C16B—H16F	0.9600
C17A—C18A	1.504 (2)	C17B—C18B	1.506 (3)
C17A—H17A	0.9700	C17B—H17C	0.9700
C17A—H17B	0.9700	C17B—H17D	0.9700
C18A—C23A	1.376 (3)	C18B—C19B	1.376 (3)
C18A—C19A	1.390 (2)	C18B—C23B	1.391 (3)
C19A—C20A	1.375 (3)	C19B—C20B	1.384 (3)
C19A—H19A	0.9300	C19B—H19B	0.9300
C20A—C21A	1.380 (3)	C20B—C21B	1.372 (3)
C20A—H20A	0.9300	C20B—H20B	0.9300
C21A—O3A	1.366 (2)	C21B—O3B	1.367 (2)
C21A—C22A	1.376 (3)	C21B—C22B	1.382 (3)
C22A—C23A	1.383 (3)	C22B—C23B	1.372 (3)
C22A—H22A	0.9300	C22B—H22B	0.9300
C23A—H23A	0.9300	C23B—H23B	0.9300
C24A—O3A	1.415 (3)	C24B—O3B	1.412 (3)
C24A—H24A	0.9600	C24B—H24D	0.9600
C24A—H24B	0.9600	C24B—H24E	0.9600
C24A—H24C	0.9600	C24B—H24F	0.9600
O1A—S1A	1.4192 (14)	O1B—S1B	1.4181 (14)
O2A—S1A	1.4213 (14)	O2B—S1B	1.4197 (14)
			
C5A—N1A—C2A	107.88 (13)	C5B—N1B—C2B	107.57 (14)
C5A—N1A—S1A	123.21 (11)	C5B—N1B—S1B	123.08 (12)
C2A—N1A—S1A	126.23 (12)	C2B—N1B—S1B	125.78 (12)
C3A—C2A—N1A	108.60 (15)	C3B—C2B—N1B	108.71 (15)
C3A—C2A—C16A	127.44 (17)	C3B—C2B—C16B	127.32 (17)
N1A—C2A—C16A	123.93 (17)	N1B—C2B—C16B	123.92 (17)
C2A—C3A—C4A	108.57 (15)	C2B—C3B—C4B	108.57 (16)
C2A—C3A—C17A	125.91 (17)	C2B—C3B—C17B	125.76 (17)
C4A—C3A—C17A	125.51 (16)	C4B—C3B—C17B	125.67 (17)
C9A—C4A—C5A	119.20 (16)	C9B—C4B—C5B	119.45 (17)
C9A—C4A—C3A	132.64 (16)	C9B—C4B—C3B	132.38 (18)
C5A—C4A—C3A	108.16 (15)	C5B—C4B—C3B	108.17 (15)
C6A—C5A—C4A	122.07 (16)	C6B—C5B—C4B	121.90 (17)
C6A—C5A—N1A	131.23 (15)	C6B—C5B—N1B	131.17 (17)
C4A—C5A—N1A	106.70 (14)	C4B—C5B—N1B	106.90 (14)
C5A—C6A—C7A	117.48 (17)	C7B—C6B—C5B	117.0 (2)
C5A—C6A—H6A	121.3	C7B—C6B—H6B	121.5
C7A—C6A—H6A	121.3	C5B—C6B—H6B	121.5
C6A—C7A—C8A	121.15 (18)	C8B—C7B—C6B	121.8 (2)
C6A—C7A—H7A	119.4	C8B—C7B—H7B	119.1
C8A—C7A—H7A	119.4	C6B—C7B—H7B	119.1
C9A—C8A—C7A	121.13 (18)	C9B—C8B—C7B	121.3 (2)
C9A—C8A—H8A	119.4	C9B—C8B—H8B	119.4
C7A—C8A—H8A	119.4	C7B—C8B—H8B	119.4
C8A—C9A—C4A	118.97 (17)	C8B—C9B—C4B	118.5 (2)
C8A—C9A—H9A	120.5	C8B—C9B—H9B	120.7
C4A—C9A—H9A	120.5	C4B—C9B—H9B	120.7
C11A—C10A—C15A	121.56 (16)	C11B—C10B—C15B	121.21 (17)
C11A—C10A—S1A	119.34 (13)	C11B—C10B—S1B	119.68 (13)
C15A—C10A—S1A	119.10 (13)	C15B—C10B—S1B	119.10 (14)
C12A—C11A—C10A	118.55 (18)	C12B—C11B—C10B	119.37 (18)
C12A—C11A—H11A	120.7	C12B—C11B—H11B	120.3
C10A—C11A—H11A	120.7	C10B—C11B—H11B	120.3
C11A—C12A—C13A	120.43 (19)	C13B—C12B—C11B	119.9 (2)
C11A—C12A—H12A	119.8	C13B—C12B—H12B	120.1
C13A—C12A—H12A	119.8	C11B—C12B—H12B	120.1
C14A—C13A—C12A	120.55 (19)	C14B—C13B—C12B	120.6 (2)
C14A—C13A—H13A	119.7	C14B—C13B—H13B	119.7
C12A—C13A—H13A	119.7	C12B—C13B—H13B	119.7
C13A—C14A—C15A	120.16 (19)	C13B—C14B—C15B	120.6 (2)
C13A—C14A—H14A	119.9	C13B—C14B—H14B	119.7
C15A—C14A—H14A	119.9	C15B—C14B—H14B	119.7
C14A—C15A—C10A	118.75 (17)	C14B—C15B—C10B	118.32 (19)
C14A—C15A—H15A	120.6	C14B—C15B—H15B	120.8
C10A—C15A—H15A	120.6	C10B—C15B—H15B	120.8
C2A—C16A—H16A	109.5	C2B—C16B—H16D	109.5
C2A—C16A—H16B	109.5	C2B—C16B—H16E	109.5
H16A—C16A—H16B	109.5	H16D—C16B—H16E	109.5
C2A—C16A—H16C	109.5	C2B—C16B—H16F	109.5
H16A—C16A—H16C	109.5	H16D—C16B—H16F	109.5
H16B—C16A—H16C	109.5	H16E—C16B—H16F	109.5
C18A—C17A—C3A	115.02 (15)	C3B—C17B—C18B	113.98 (15)
C18A—C17A—H17A	108.5	C3B—C17B—H17C	108.8
C3A—C17A—H17A	108.5	C18B—C17B—H17C	108.8
C18A—C17A—H17B	108.5	C3B—C17B—H17D	108.8
C3A—C17A—H17B	108.5	C18B—C17B—H17D	108.8
H17A—C17A—H17B	107.5	H17C—C17B—H17D	107.7
C23A—C18A—C19A	117.21 (16)	C19B—C18B—C23B	117.28 (17)
C23A—C18A—C17A	121.64 (17)	C19B—C18B—C17B	121.71 (17)
C19A—C18A—C17A	121.14 (17)	C23B—C18B—C17B	121.01 (17)
C20A—C19A—C18A	121.41 (17)	C18B—C19B—C20B	122.09 (17)
C20A—C19A—H19A	119.3	C18B—C19B—H19B	119.0
C18A—C19A—H19A	119.3	C20B—C19B—H19B	119.0
C19A—C20A—C21A	120.03 (17)	C21B—C20B—C19B	119.55 (17)
C19A—C20A—H20A	120.0	C21B—C20B—H20B	120.2
C21A—C20A—H20A	120.0	C19B—C20B—H20B	120.2
O3A—C21A—C22A	124.57 (17)	O3B—C21B—C20B	125.15 (17)
O3A—C21A—C20A	115.69 (16)	O3B—C21B—C22B	115.42 (16)
C22A—C21A—C20A	119.73 (17)	C20B—C21B—C22B	119.43 (17)
C21A—C22A—C23A	119.22 (18)	C23B—C22B—C21B	120.35 (17)
C21A—C22A—H22A	120.4	C23B—C22B—H22B	119.8
C23A—C22A—H22A	120.4	C21B—C22B—H22B	119.8
C18A—C23A—C22A	122.29 (18)	C22B—C23B—C18B	121.26 (17)
C18A—C23A—H23A	118.9	C22B—C23B—H23B	119.4
C22A—C23A—H23A	118.9	C18B—C23B—H23B	119.4
O3A—C24A—H24A	109.5	O3B—C24B—H24D	109.5
O3A—C24A—H24B	109.5	O3B—C24B—H24E	109.5
H24A—C24A—H24B	109.5	H24D—C24B—H24E	109.5
O3A—C24A—H24C	109.5	O3B—C24B—H24F	109.5
H24A—C24A—H24C	109.5	H24D—C24B—H24F	109.5
H24B—C24A—H24C	109.5	H24E—C24B—H24F	109.5
C21A—O3A—C24A	117.59 (16)	C21B—O3B—C24B	117.74 (17)
O1A—S1A—O2A	119.34 (9)	O1B—S1B—O2B	119.31 (9)
O1A—S1A—N1A	106.26 (8)	O1B—S1B—N1B	106.45 (8)
O2A—S1A—N1A	107.20 (8)	O2B—S1B—N1B	106.90 (8)
O1A—S1A—C10A	108.19 (8)	O1B—S1B—C10B	108.35 (8)
O2A—S1A—C10A	108.93 (8)	O2B—S1B—C10B	109.11 (8)
N1A—S1A—C10A	106.17 (7)	N1B—S1B—C10B	105.93 (7)
			
C5A—N1A—C2A—C3A	2.93 (18)	C5B—N1B—C2B—C3B	2.57 (18)
S1A—N1A—C2A—C3A	164.68 (12)	S1B—N1B—C2B—C3B	161.65 (12)
C5A—N1A—C2A—C16A	−179.01 (16)	C5B—N1B—C2B—C16B	−179.91 (16)
S1A—N1A—C2A—C16A	−17.3 (2)	S1B—N1B—C2B—C16B	−20.8 (2)
N1A—C2A—C3A—C4A	−1.80 (18)	N1B—C2B—C3B—C4B	−1.20 (19)
C16A—C2A—C3A—C4A	−179.77 (17)	C16B—C2B—C3B—C4B	−178.62 (17)
N1A—C2A—C3A—C17A	179.27 (15)	N1B—C2B—C3B—C17B	178.69 (15)
C16A—C2A—C3A—C17A	1.3 (3)	C16B—C2B—C3B—C17B	1.3 (3)
C2A—C3A—C4A—C9A	−179.35 (17)	C2B—C3B—C4B—C9B	178.77 (19)
C17A—C3A—C4A—C9A	−0.4 (3)	C17B—C3B—C4B—C9B	−1.1 (3)
C2A—C3A—C4A—C5A	−0.01 (18)	C2B—C3B—C4B—C5B	−0.62 (19)
C17A—C3A—C4A—C5A	178.93 (15)	C17B—C3B—C4B—C5B	179.49 (16)
C9A—C4A—C5A—C6A	0.7 (2)	C9B—C4B—C5B—C6B	1.1 (3)
C3A—C4A—C5A—C6A	−178.74 (14)	C3B—C4B—C5B—C6B	−179.45 (15)
C9A—C4A—C5A—N1A	−178.75 (14)	C9B—C4B—C5B—N1B	−177.29 (15)
C3A—C4A—C5A—N1A	1.80 (17)	C3B—C4B—C5B—N1B	2.18 (18)
C2A—N1A—C5A—C6A	177.74 (16)	C2B—N1B—C5B—C6B	178.96 (17)
S1A—N1A—C5A—C6A	15.3 (2)	S1B—N1B—C5B—C6B	19.2 (3)
C2A—N1A—C5A—C4A	−2.87 (17)	C2B—N1B—C5B—C4B	−2.89 (18)
S1A—N1A—C5A—C4A	−165.29 (11)	S1B—N1B—C5B—C4B	−162.66 (12)
C4A—C5A—C6A—C7A	−0.6 (2)	C4B—C5B—C6B—C7B	−0.6 (3)
N1A—C5A—C6A—C7A	178.69 (16)	N1B—C5B—C6B—C7B	177.29 (17)
C5A—C6A—C7A—C8A	0.4 (3)	C5B—C6B—C7B—C8B	−0.1 (3)
C6A—C7A—C8A—C9A	−0.2 (3)	C6B—C7B—C8B—C9B	0.3 (4)
C7A—C8A—C9A—C4A	0.3 (3)	C7B—C8B—C9B—C4B	0.1 (3)
C5A—C4A—C9A—C8A	−0.5 (2)	C5B—C4B—C9B—C8B	−0.8 (3)
C3A—C4A—C9A—C8A	178.78 (17)	C3B—C4B—C9B—C8B	179.87 (19)
C15A—C10A—C11A—C12A	0.2 (2)	C15B—C10B—C11B—C12B	0.5 (3)
S1A—C10A—C11A—C12A	179.03 (14)	S1B—C10B—C11B—C12B	179.83 (15)
C10A—C11A—C12A—C13A	−0.3 (3)	C10B—C11B—C12B—C13B	−0.1 (3)
C11A—C12A—C13A—C14A	0.2 (3)	C11B—C12B—C13B—C14B	−0.2 (4)
C12A—C13A—C14A—C15A	−0.1 (3)	C12B—C13B—C14B—C15B	0.1 (4)
C13A—C14A—C15A—C10A	0.1 (3)	C13B—C14B—C15B—C10B	0.4 (3)
C11A—C10A—C15A—C14A	−0.2 (2)	C11B—C10B—C15B—C14B	−0.7 (3)
S1A—C10A—C15A—C14A	−178.95 (13)	S1B—C10B—C15B—C14B	−179.95 (15)
C2A—C3A—C17A—C18A	−85.4 (2)	C2B—C3B—C17B—C18B	−82.4 (2)
C4A—C3A—C17A—C18A	95.9 (2)	C4B—C3B—C17B—C18B	97.4 (2)
C3A—C17A—C18A—C23A	116.8 (2)	C3B—C17B—C18B—C19B	120.4 (2)
C3A—C17A—C18A—C19A	−64.6 (2)	C3B—C17B—C18B—C23B	−60.4 (3)
C23A—C18A—C19A—C20A	2.5 (3)	C23B—C18B—C19B—C20B	−0.6 (3)
C17A—C18A—C19A—C20A	−176.14 (18)	C17B—C18B—C19B—C20B	178.61 (17)
C18A—C19A—C20A—C21A	−0.1 (3)	C18B—C19B—C20B—C21B	−0.6 (3)
C19A—C20A—C21A—O3A	177.53 (17)	C19B—C20B—C21B—O3B	−178.41 (17)
C19A—C20A—C21A—C22A	−2.8 (3)	C19B—C20B—C21B—C22B	2.0 (3)
O3A—C21A—C22A—C23A	−177.08 (18)	O3B—C21B—C22B—C23B	178.21 (17)
C20A—C21A—C22A—C23A	3.3 (3)	C20B—C21B—C22B—C23B	−2.2 (3)
C19A—C18A—C23A—C22A	−2.0 (3)	C21B—C22B—C23B—C18B	0.9 (3)
C17A—C18A—C23A—C22A	176.63 (18)	C19B—C18B—C23B—C22B	0.5 (3)
C21A—C22A—C23A—C18A	−0.9 (3)	C17B—C18B—C23B—C22B	−178.77 (18)
C22A—C21A—O3A—C24A	5.2 (3)	C20B—C21B—O3B—C24B	7.0 (3)
C20A—C21A—O3A—C24A	−175.18 (18)	C22B—C21B—O3B—C24B	−173.44 (18)
C5A—N1A—S1A—O1A	−40.19 (15)	C5B—N1B—S1B—O1B	−41.21 (15)
C2A—N1A—S1A—O1A	160.69 (14)	C2B—N1B—S1B—O1B	162.76 (14)
C5A—N1A—S1A—O2A	−168.85 (13)	C5B—N1B—S1B—O2B	−169.76 (13)
C2A—N1A—S1A—O2A	32.03 (16)	C2B—N1B—S1B—O2B	34.21 (17)
C5A—N1A—S1A—C10A	74.84 (14)	C5B—N1B—S1B—C10B	73.98 (15)
C2A—N1A—S1A—C10A	−84.28 (15)	C2B—N1B—S1B—C10B	−82.05 (15)
C11A—C10A—S1A—O1A	−159.14 (13)	C11B—C10B—S1B—O1B	−160.15 (14)
C15A—C10A—S1A—O1A	19.67 (15)	C15B—C10B—S1B—O1B	19.15 (16)
C11A—C10A—S1A—O2A	−27.99 (15)	C11B—C10B—S1B—O2B	−28.80 (16)
C15A—C10A—S1A—O2A	150.82 (13)	C15B—C10B—S1B—O2B	150.50 (14)
C11A—C10A—S1A—N1A	87.15 (14)	C11B—C10B—S1B—N1B	85.96 (15)
C15A—C10A—S1A—N1A	−94.04 (13)	C15B—C10B—S1B—N1B	−94.74 (15)

### Hydrogen-bond geometry (Å, °)

**Table:**

*D*—H···*A*	*D*—H	H···*A*	*D*···*A*	*D*—H···*A*
C11A—H11A···O1B^i^	0.93	2.52	3.397 (2)	157
C11B—H11B···O1A^ii^	0.93	2.49	3.337 (2)	151
C15A—H15A···O3B^iii^	0.93	2.53	3.398 (2)	155

Symmetry codes: (i) −*x*+1, −*y*+1, −*z*+1; (ii) −*x*, −*y*+1, −*z*+1; (iii) *x*−1, *y*−1, *z*+1.
